# Long insert whole genome sequencing for copy number variant and translocation detection

**DOI:** 10.1093/nar/gkt865

**Published:** 2013-09-25

**Authors:** Winnie S. Liang, Jessica Aldrich, Waibhav Tembe, Ahmet Kurdoglu, Irene Cherni, Lori Phillips, Rebecca Reiman, Angela Baker, Glen J. Weiss, John D. Carpten, David W. Craig

**Affiliations:** ^1^Integrated Cancer Genomics Division, Translational Genomics Research Institute, Phoenix, AZ, 85004, USA, ^2^Neurogenomics Division, Translational Genomics Research Institute, Phoenix, AZ, 85004, USA and ^3^Cancer Treatment Centers of America, Medical Oncology, Goodyear, AZ, 85338, USA

## Abstract

As next-generation sequencing continues to have an expanding presence in the clinic, the identification of the most cost-effective and robust strategy for identifying copy number changes and translocations in tumor genomes is needed. We hypothesized that performing shallow whole genome sequencing (WGS) of 900–1000-bp inserts (long insert WGS, LI-WGS) improves our ability to detect these events, compared with shallow WGS of 300–400-bp inserts. *A priori* analyses show that LI-WGS requires less sequencing compared with short insert WGS to achieve a target physical coverage, and that LI-WGS requires less sequence coverage to detect a heterozygous event with a power of 0.99. We thus developed an LI-WGS library preparation protocol based off of Illumina’s WGS library preparation protocol and illustrate the feasibility of performing LI-WGS. We additionally applied LI-WGS to three separate tumor/normal DNA pairs collected from patients diagnosed with different cancers to demonstrate our application of LI-WGS on actual patient samples for identification of somatic copy number alterations and translocations. With the evolution of sequencing technologies and bioinformatics analyses, we show that modifications to current approaches may improve our ability to interrogate cancer genomes.

## INTRODUCTION

Next-generation sequencing (NGS) has allowed for the rapid characterization of genomes, exomes and transcriptomes. Such advances have been applied to personalized oncology, represent a promising approach for identifying therapeutic options for cancer patients who do not respond to standard treatments and are key to improving our understanding of tumorigenesis (1–3). However, although the cost of performing whole genome sequencing (WGS) has decreased in recent years, it is more costly compared with exome and RNA sequencing (RNAseq) when sequencing to 30× coverage. Owing to this caveat and the existing utility of using deep exome sequencing to identify potentially targetable small somatic events in cancer genomes, the need for identifying an alternative WGS strategy for identifying breakpoints, which characterize structural variants and copy number changes, is clear.

One option for evaluating larger regions in whole genome data using sequencing by synthesis (SBS) technology is the use of Illumina’s mate pair library preparation protocol. The standard protocol requires 10 µg of genomic DNA and supports the evaluation of regions spanning up to ∼2–5 kb. However, owing to the limited amount of DNA that is typically available from tumor biopsies, this approach is not a viable option for sequencing. Illumina also recently released a new Nextera Mate Pair Sample Preparation Kit that requires 1–4 µg of genomic DNA. However, this approach retains transposome-mediated fragmentation that results in an enzymatic footprint that requires trimming of sequencing data, and still requires circularization and biotin pull-down, and thus decreases the ease of library preparation. An alternative user-friendly strategy that requires lower inputs, that does not require post-sequencing trimming and that allows for increased physical coverage and analysis of regions greater than that accomplished by short insert (SI) sequencing is thus needed.

We hypothesized that performing shallow WGS using longer inserts that are ∼900–1000-bp long increases our power for identifying breakpoints, and thereby copy number alterations and translocations, compared with shallow SI WGS of 300–400-bp inserts, which was used by the MI-ONCOSEQ (Michigan Oncology Sequencing Project) study as the solution for identifying structural variants and copy number changes (3). Previous research using alternative methods has also shown that our ability to identify breakpoints is increased when sequencing longer inserts (4). In this study, we first tested our hypothesis using *a priori* analyses, and subsequently developed a long insert (LI) whole genome library preparation protocol that retains the entire insert in the final library, as opposed to mate pair protocols that enzymatically remove central insert sequences. We then applied this approach to tumor/normal DNA pairs collected from three separate patients diagnosed with different malignancies including metastatic basal cell carcinoma of the skin, metastatic papillary renal carcinoma and metastatic bronchial neuroendocrine cancer. We demonstrate both the feasibility of LI-WGS and its application in simultaneously identifying copy number alterations and translocations, key events that characterize cancer genomes.

## MATERIALS AND METHODS

### Modeling the relationship between physical coverage and insert size

To evaluate the relationship between insert size and physical coverage, we outlined a model for determining physical coverage. Physical coverage can be calculated by using the following equation (5):

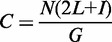

  where *C* = physical coverage
      *N* = number of aligned reads
      *L* = read length (a multiplier of 2 is used for paired-end (PE) sequencing)
      *G* = size of human genome
       *I* = inter-read base pair (bp) distance for PE sequencing such that the insert size equals *2L + I*

The earlier equation can be condensed to the following:

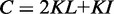

  where 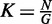

Because the approximate number of aligned reads is typically consistent across human genomes for a given aligner and the size of the human genome does not change, we treat *K* as a constant value. Physical coverage increases as the distance between reads increases.

### Power analysis

We performed power analyses using the following equation:

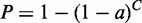

  Where *P* = power
       *a* = frequency of event
       *C* = physical coverage/number of anomalous reads

### Protocol optimization

Development and optimization of LI whole genome library preparation was performed using Roche human genomic DNA (catalog# 11691112001) and Illumina’s TruSeq DNA Sample Prep Kit (TruSeq DNA Sample Preparation v2 Guide, Part 15026486 Revision A). The final protocol is as follows:

- Fragmentation—For each sample 1.1 µg of DNA was fragmented on the Covaris E210 to a target size of 900–1000 bp (Duty cycle: 2%, Intensity: 6, Cycles/burst: 200, Time: 20 s, Temperature: 4°C). 100 ng of the sample was run on a 1% Tris acetate EDTA (TAE) gel to verify fragmentation.
- End repair—This step is performed according to the manufacturer’s protocol.
- End repair purification—100 µl of AMPure XP beads were added directly to end repair products for purification. A 1:1 bead volume:sample volume is used and 300 µl of 80% ethanol was used for two total washes. Aside from these exceptions, the manufacturer’s protocol was followed.
- Adenylation and ligation—These steps are performed according to the manufacturer’s protocol.
- Ligation purification—42.5 µl nuclease-free water is used to resuspend the dried bead pellet. Following mixing, a 2 min incubation at room temperature and a 2 min incubation on a magnet, 40 µl of supernatant is aspirated for ligation. Steps 14–26 are removed from the ‘Clean up ALP’ step in the manufacturer’s protocol.
- Size selection—A 400 ml 1.5% TAE gel is used for size selection instead of a 150 ml 2% gel. Multiple gel punches can be taken. Punches are placed in separate Bio-Rad Freeze ‘N Squeeze columns for purification. Columns are placed at −20°C for 5 min and centrifuged at maximum speed for 3 min—this process is performed 5×. The final eluate is purified using AMPure beads. The same purification protocol is used as is described in the ‘Clean up IMP’ section (under ‘Perform End Repair’) with the following minor edits: a 1:1 ratio of bead volume:sample volume is used during purification, the final sample is resuspended in 22.5 µl nuclease-free water and 20 µl of the supernatant is used for enrichment polymerase chain reaction (PCR).
- Enrichment PCR—A modified PCR is used:
  1. 98°C for 30 s
  2. 98°C for 10 s
  3. 60°C for 30 s
  4. 72°C for 1 min
  5. Cycle to step 2 eight more times
  6. 72°C for 10 min
  7. 4°C hold
  8. PCR purification—The manufacturer’s protocol is followed for this step aside from the following: 40 µl beads are added to the PCR product for a 1:1 sample to bead ratio, the final dried pellet is resuspended in 26.5 µl resuspension buffer and 25 µl of the final supernatant is removed as the final library.

Final libraries were quantified by Qubit and library sizes determined using the Agilent Bioanalyzer. The LI test library was clustered and sequenced on a single flowcell lane on the Illumina HiSeq to evaluate clustering efficiency. Based on the total and pass filter (PF) cluster densities, the loaded library concentration was adjusted to 18–20 pM for future samples.

### Patient sample assessment

The study was conducted in accordance with the Declaration of Helsinki and was approved by the Western Institutional Review Board (Protocol #20101288) (NCT01443390). Patients must be age ≥18 and willing to undergo a biopsy or surgical procedure to obtain tissue, unless a frozen tumor collected <8 weeks prior was available. Interested participants were made aware that obtaining a new biopsy may not be a part of the patient’s routine care for their malignancy. Other eligibility criteria included baseline laboratory data indicating acceptable bone marrow reserve, liver and renal function, Karnofsky performance status ≥80% and life expectancy >3 months. All eligible patients had fresh frozen tumor sample collected and sent for analyses. Normal DNA was obtained from peripheral blood mononuclear cells. Direct visualization of patient 1 and 2’s was performed by a board certified pathologist to determine tumor cellularity.

### Genomic DNA isolation

Tissue was disrupted and homogenized in RNeasy lysis buffer (Buffer RLT) plus (Qiagen AllPrep DNA/RNA Mini Kit) using the Bullet Blender™ and transferred to a tube containing Buffer RLT plus and stainless steel beads. Blood leukocytes were isolated from whole blood by centrifugation at room temperature and resuspended in Buffer RLT plus. All samples were homogenized and centrifuged, and DNA were isolated following the AllPrep protocol. Each sample was evaluated by gel electrophoresis, analyzed using the Nanodrop to evaluate absorbance ratios and quantified using Invitrogen’s Qubit Fluorometer.

### SI and LI whole genome library preparation

1.1 µg genomic DNA of each sample was used to create SI whole genome libraries using Illumina’s TruSeq DNA Sample Kit per manufacturer’s protocol. One modification is that size-selected products were purified using Bio-Rad Freeze ‘N Squeeze gel purification columns and AMPure XP beads. Products were PCR enriched and purified following the manufacturer’s protocol. LI libraries were prepared and indexed using Illumina’s TruSeq DNA Sample Kit with modifications listed previously. Final libraries were quantified and library sizes determined using the Bioanalyzer and Qubit.

### Exome library preparation for copy number validation

Exome libraries were prepared using 3 µg of genomic DNA for the same tumor and normal samples that were whole genome sequenced. Genomic DNA was fragmented to an approximate target size of 150–200 bp on the Covaris E210. For each sample, 100 ng of each fragmented product was run on 2% TAE gel to verify fragmentation. Library preparation was performed using New England Biolab’s (NEB) NEBNext DNA Sample Prep Master Mix Kit, Illumina Multiplexing Oligonucleotide Kit, Agilent SureSelect Human All Exon 50 Mb Kit and Agilent Herculase II Fusion DNA Polymerase. End repair was performed using NEBNext End Repair Buffer (10×), End Repair Enzyme Mix and the fragmented DNA samples. End repair products were purified using AMPure XP beads: 180 µl of resuspended beads were used for cleaning each sample, two 70% ethanol washes were performed and samples were dried for 20 min at room temperature before resuspension in 44 µl of warm elution buffer. For each sample, 42 µl of cleaned end repaired samples are input into adenylation which was performed using NEBNext dA-tailing Buffer (10×) and NEBNext Klenow fragment (3′→5′ exo). Adenylated products were cleaned using AMPure XP beads as previously described but 90 µl of beads are used for cleaning and the final samples are eluded with 15 µl of nuclease-free water. Each adenylated sample was used for indexed adapter ligation. This step is performed using the NEBNext Ligation Buffer (5×), NEBNext T4 ligase and Index PE adapter oligonucleotide mix from Illumina’s Multiplexing Oligonucleotide Kit. Reactions were purified using AMPure XP beads and enrichment PCR was performed using InPE1.0 forward PCR primer (Illumina Multiplexing Oligonucleotide Kit), SureSelect Indexing Pre-cap PCR primer, Herculase II 5× reaction buffer, Herculase dNTP mix and Herculase II polymerase. The following PCR program was used:

1. 98°C for 2 min
2. 98°C for 20 s
3. 65°C for 30 s
4. 72°C for 30 s
5. Cycle to step 2 five more times
6. 72°C for 5 min
7. 4°C hold

PCR products were purified using AMPure XP beads. Each sample was run on the Agilent Bioanalyzer using the Agilent DNA 1000 assay and quantified using the Qubit. 500 ng of each sample was used for capture. From hybridization onward, Agilent’s SureSelect Target Enrichment System for Illumina Paired-End Multiplexed Sequencing protocol (version 1.2) was followed.

### PE sequencing

Libraries were used to generate clusters on HiSeq Paired End v3 flowcells on the Illumina cBot using Illumina’s TruSeq PE Cluster Kit v3. One exception is that for patient 1, three lanes of SI normal and three lanes of SI tumor whole genomes were sequenced on a v1.5 flowcell. Clustered flowcells were sequenced on the Illumina HiSeq 2000 using Illumina’s TruSeq SBS Kit. Each LI WG library was run in a single lane, and tumor/normal exome pools were sequenced in individual lanes.

### Sequencing data analysis

Raw sequence data were converted to fastq files using Illumina’s BCLConverter. Fastq files were validated to evaluate the distribution of quality scores and to ensure that quality scores do not drastically drop over each read. Validated fastq files for whole genome and exome data were aligned to the human reference genome (build 37) using the Burrows–Wheeler Alignment tool (6) and sorted with SAMtools (7) to create binary sequence (bam) files. Lane level bam files were indel realigned and recalibrated using Genome Analysis Toolkit (8). Lane level bam files were then merged as necessary and PCR duplicates were flagged for removal using Picard (http://picard.sourceforge.net), which was also used to evaluate GC metrics.

To compare across SI and LI data, SAMtools was used to randomly select 250 million mapped reads from each data set, and these reads were saved as ‘normalized’ bam’s. To detect translocations in SI and LI normalized data, we first defined the range of insert sizes in the normal data, evaluated the tumor data using a window size that is 3× the insert size range of the normal data and identified reads in each window that maps to a different location. A minimum of eight reads mapping to a discordant location was required for a translocation to be called. For this analysis, we generated a script to identify anomalous read pairs (https://github.com/davcraig75/tgen_somaticSV). To decrease false negatives, discordant locations to which at least four tumor reads map are also called. Each event was also manually inspected for confirmation. Copy number analysis was completed by determining the log2 difference of the normalized physical coverage (or clonal coverage) for both germline and tumor samples separately across a sliding 2 kb window of the mean. Our anomalous read pair script also determines the ratio of anomalous read pairs over all read pairs that mark the boundary of a copy number change. The derivative log ratio spread (DLRS) for each sample was calculated by determining the standard deviation of the point-to-point difference across the genome divided by the square root of 2. The average distance between points is 80 kb and the smoothing window is 19 kb.

### Translocation validation

Selected breakpoints were visualized using the Integrative Genomics Viewer (Broad Institute), and primers were designed to flank breakpoints using PrimerQuest (Integrated DNA Technologies). Primers were used to PCR amplify regions encompassing breakpoints on the same DNA samples that were sequenced. PCR products were Sanger sequenced to confirm presence of breakpoints.

## RESULTS

### The utility of LI-WGS

**Figure 1.**
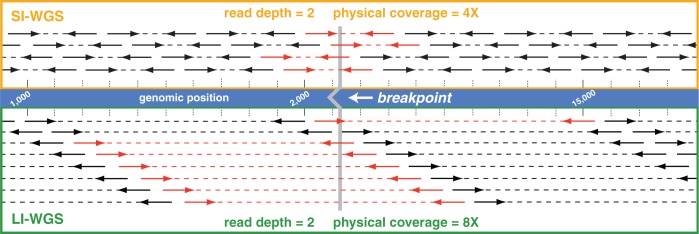
Comparison of SI- and LI-WGS. A visualization of mapped reads for SI- and LI-WGS is shown assuming a read depth of 2 for each library type. The reference human genome is shown in the middle of the figure, and the location of a theoretical breakpoint is shown in gray with the location of the breakpoint marked by the gray line. SI (300 bp) mapped reads are displayed above the reference, and LI (900 bp) mapped reads are displayed below the reference. PE reads are represented by heavy solid lines with arrowheads and regions between reads are denoted by a dotted line. Anomalous read pairs are shown in red. Higher physical coverage is achieved for LI-WGS libraries when sequencing to the same read depth for SI- and LI-WGS libraries. Furthermore, by interrogating a larger genomic region using LIs, the likelihood that a breakpoint will fall within that region is increased.

Using *a priori* analyses, we determined that physical coverage is directly affected by insert size such that physical coverage increases with longer insert sizes when sequencing a fixed read length (calculations described in Methods). Physical coverage is considered in this analysis because it reflects the size of the insert being sequenced and is associated with our ability to identify copy number variants (CNVs) and translocations. In Figure 1, we show a theoretical comparison of SI- and LI-WGS mapped reads. When sequencing to the same read depth, higher physical coverage is achieved for LI libraries (900-bp inserts) compared with SI libraries (300-bp inserts) and thereby increases our power for detecting CNVs or translocations. Theoretical anomalous read pairs are shown in red; with higher physical coverage, our ability to detect a breakpoint is increased. In addition, we capture information on a larger genomic region when sequencing LI libraries, and thus increase the likelihood that a breakpoint will fall within that region and be detected. In Supplementary Figure S1, we outline the relationship between physical coverage and the amount of sequencing that is needed to achieve a target physical coverage for SI- and LI-WGS. Overall, our simplified model shows that given a target physical coverage, more sequencing is needed for SI libraries compared with LI libraries. A few caveats of this analysis are that we did not address potential contributions from factors such as GC bias and polymerase fidelity, and we also make the assumption that read depth is evenly distributed across the entire genome although a Poisson distribution is typically observed in sequencing data. To address these caveats and to truly evaluate this relationship between physical coverage and insert size, we additionally performed experimental analyses to compare SI and LI libraries.

### LI-WGS power analyses

**Figure 2.**
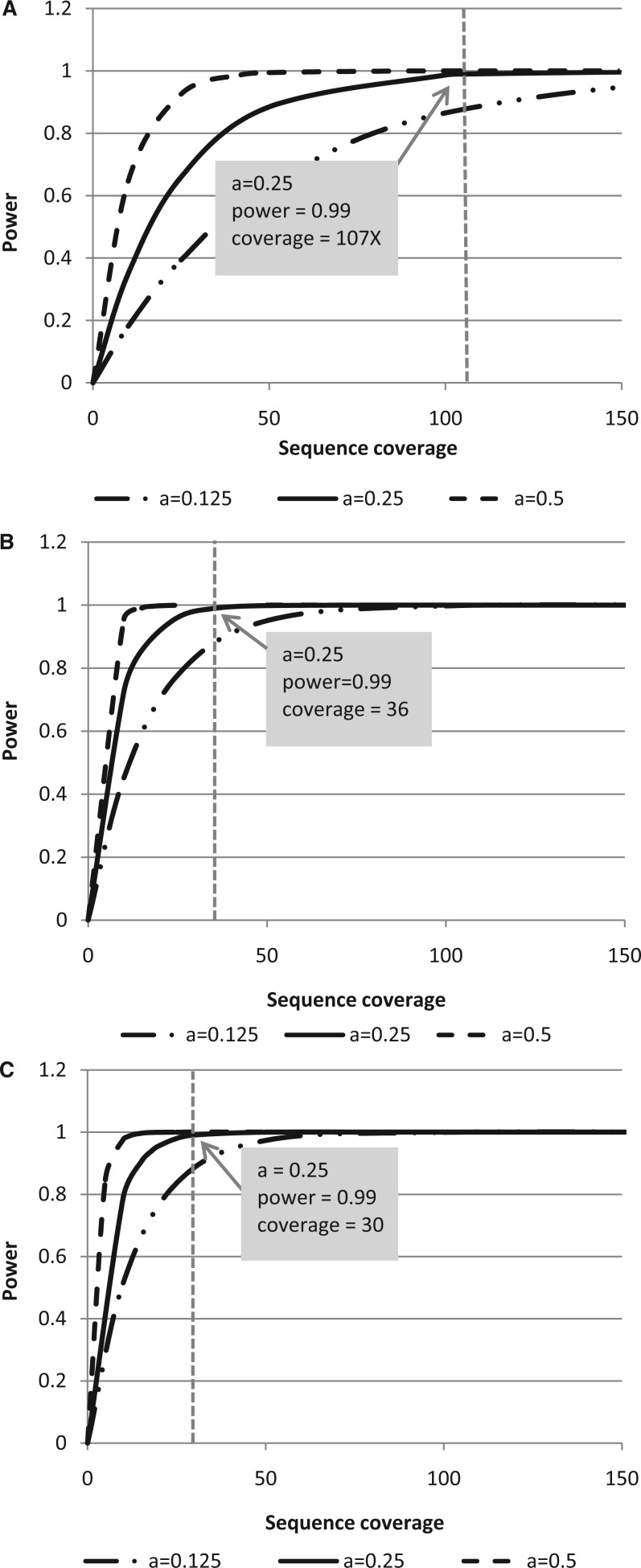
Comparison of power achieved when sequencing LI or SI libraries. Power calculations were performed to evaluate the power achieved when sequencing SI (300 bp) libraries with a 2 × 100 read length (**A**). These analyses were performed to determine the power of identifying a heterozygous somatic event as characterized by at least 10 anomalous read pairs under three scenarios where a tumor sample may have three different tumor cellularities (100, 50, 25% tumor). This analysis was similarly performed for LI (900 bp) libraries with a 2 × 100 read length (**B**). We performed additional LI analyses using the same parameters but decreased the read length from 2 × 100 to 2 × 83 (**C**). For all three analyses, a dotted line demarcates the sequence coverage needed for detecting a heterozygous event in a sample with 50% tumor cellularity and 0.99 power. Coverage shown is sequence coverage, and *a* is the expected frequency of an event given the different tumor cellularities.

Power calculations were performed to evaluate the amount of sequence coverage that is needed to detect a structural variant in differently sized inserts. Figure 2A and B show a comparison of achieved power when sequencing 300-bp inserts or 900-bp inserts where *a* is the frequency of the somatic event. Three mutation frequencies were evaluated to consider three scenarios in which the tumor cell content of the analyzed sample is 100, 50 or 25% tumor. We assumed that the event is heterogeneous such that the expected frequency of an event *a* is one-half of the percent tumor cellularity. We required that a minimum of 10 anomalous read pairs be needed to detect an event where an anomalous read pair is defined as one in which the mapping distance between the two ends are substantially greater than the mean inter-read distance, or if the pairs map to different chromosomes. We also performed additional power calculations and assumed a shorter read length for 900-bp insert libraries to evaluate the utility of sequencing less when longer inserts are used (2 × 83 cycle read length; Figure 2C). A 2 × 83 read length was selected based on the format of Illumina’s sequencing reagents as three 50 cycle kits can be used to perform approximately a 2 × 83 sequencing run.

Using SI libraries and assuming 50% tumor cellularity, 107× sequence coverage (161× physical coverage) is needed to achieve 0.99 power for detecting 10 anomalous read pairs. However, when sequencing a 900-bp insert under the same conditions and sequencing shorter read lengths, only 30× sequence coverage (163× physical coverage) is needed. These analyses demonstrate that even with shorter read lengths and less sequencing, LI-WGS using 900-bp inserts, as opposed to 300-bp inserts, increases the power of detecting an event.

### LI-WGS library preparation protocol development

Based on results from preliminary analyses, we created a LI-WGS library preparation protocol that we modified from Illumina’s TruSeq DNA Sample Prep library protocol for SI-WGS. To generate longer inserts for whole genome libraries, we modified three primary areas in Illumina’s WGS library preparation protocol: (i) fragmentation, (ii) AMPure XP bead purification steps and (iii) enrichment PCR parameters. Details on all changes to the protocol are described in the Methods and are briefly described here. We used 1.1 µg of genomic DNA for a single library preparation and following fragmentation analyzed 100 ng of fragmented product by gel electrophoresis to verify fragmentation.

**Figure 3.**
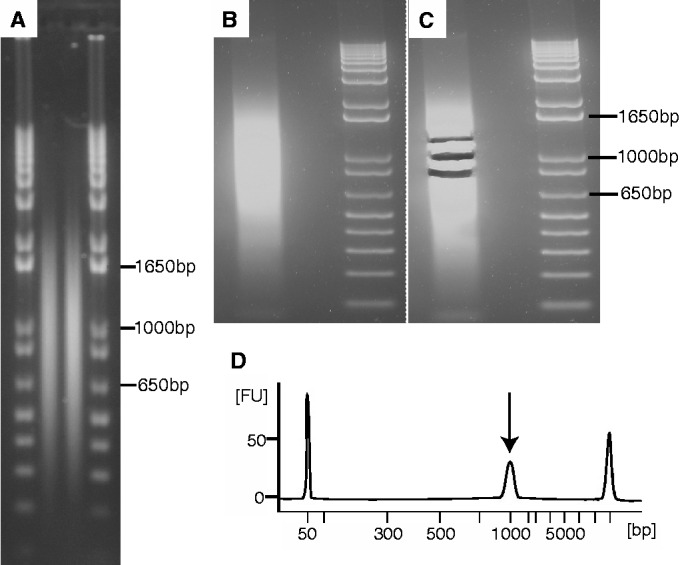
LI library preparation quality control. Two examples of fragmented human genomic samples to a target of 900 bp are shown (**A**). Fragmented samples are run alongside Invitrogen’s 1 Kb Plus DNA ladder. An example of ligation products for the LI-WGS preparation protocol is shown in (**B**). Products are run alongside the same 1 Kb Plus ladder shown in (**C**). The same gel from (B) following size selection is shown in (C) in which multiple collections of ligation product were obtained. An example Bioanalyzer trace of a final LI-WGS library is shown in (**D**; FU = fluorescence units). The library peak is demarcated by an arrow; flanking peaks are Bioanalyzer marker peaks.

During fragmentation, Illumina’s protocol for generating whole genome libraries using the TruSeq DNA Sample Prep kit fragments genomic DNA to a target size of 300–400 bp. To generate LI libraries, Covaris parameters for sonic fragmentation were modified to generate fragments that are approximately 900–1000 bp. An example of LI fragmentation products, electrophoretically separated on a 1% TAE gel, is shown in Figure 3A.The AMPure XP bead purification step following end repair was also modified with respect to the bead volume:DNA volume ratio to remove shorter molecules that are approximately 200 bp and smaller. A 1:1 bead volume:DNA volume ratio was used, and this purification was also added to the protocol following size selection. Figure 3B shows an example size selection gel for which ligation products were separated on a 1.5% TAE gel, and Figure 3C shows the post size-selection gel, after collecting 800, 1000 and 1300 bp fragments. An Agilent Bioanalyzer DNA 12000 trace illustrating the final library (size selected at 1000 bp) for a LI-WGS library preparation is shown (Figure 3D). Surveying 37 LI-WGS libraries, the median yield for this LI library preparation is 138.2 ng (6820 pM).

### Comparison of SI- and LI-WGS

With LI-WGS, the increased size of the inserts was expected to cause differences with respect to GC dropout, normalized coverage across GC rich regions, clustering efficiency, cluster size and Q30 scores. We compared an example LI-WGS library prepared according to our modified protocol and an example SI-WGS library prepared according to Illumina’s TruSeq DNA Sample Prep protocol. Sequencing each library in a single flowcell lane, we were able to achieve similar cluster densities but obtained a lower PF density with LI libraries, and thus, a lower number of PF reads. Results from the comparison are shown in Table 1. GC and AT dropout values were higher in the LI library compared with the SI library, whereas the median GC normalized coverage for the LI library was 0.76 compared with 0.86 for the SI library. The GC and AT dropout values, which can range from 0 to 100, are a measure of how much coverage is lost in GC, or AT, rich regions, respectively. GC normalized coverage is a measure of the amount of coverage that is obtained in each GC bin, as determined by Picard, divided by the mean coverage of all bins. Median GC normalized coverage values closer to one are indicative of consistent coverage over GC rich regions.

**Table 1.** Sequencing metric comparison of SI and LI libraries

Metric	SI whole genome library	LI whole genome library
Median insert size (bp)	322	869
Mean insert size (bp)	313.90	869.34
Insert size standard deviation	48.50	64.19
Number of lanes sequenced	1	1
Total cluster density (K/mm^2^)	801 ± 70	798 ± 61
PF cluster density (K/mm^2^)	91.5 ± 2.0	81.9 ± 4.8
Read length	2 × 104	2 × 83
Total reads (M)	221.56	220.59
PF reads (M)	202.48	180.28
Read 1 error rate	0.28 ± 0.03	0.43 ± 0.05
Read 2 error rate	0.48 ± 0.12	0.50 ± 0.12
Read 1 phasing/prephasing	0.136/0.201	0.184/0.252
Read 2 phasing/prephasing	0.145/0.193	0.183/0.268
Total yield (Gb)	33.11	31.12
Total Q30 yield (Gb)	29.30	25.30
%Q30	88.50	81.30
Total reads	404 968 194	360 562 104
Total mapped reads	379 311 244	335 823 767
% reads mapped	93.66	93.14
GC dropout	2.91	5.69
AT dropout	1.22	2.45
Median GC normalized coverage	0.86	0.76
Mapped sequence coverage	12.57	8.78
Mapped physical coverage	37.95	93.06

**Figure 4.**
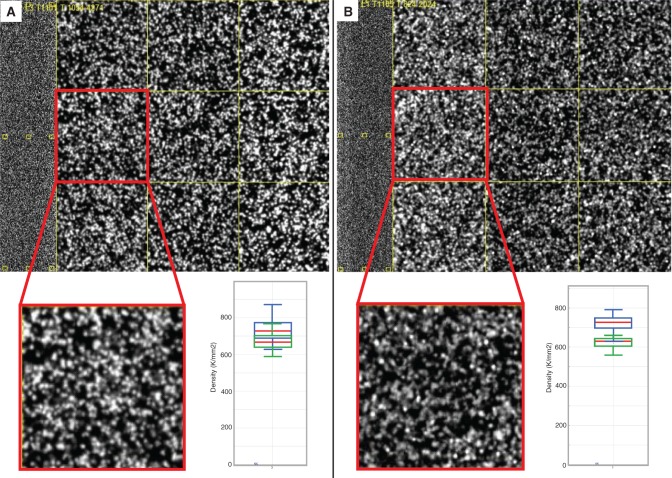
Comparison of cluster sizes between SI and LI libraries. An example image from sequencing a SI library is shown in (**A**), along with a cluster density plot from Illumina’s Sequence Analysis Viewer. An example image and cluster density plot from sequencing a LI library is shown in (**B**). In each cluster density plot, the blue boxes represent total densities and the green boxes represent PF cluster densities. Red lines demarcate the median for the total density and the PF density.

Although fewer clusters and less data are acquired when sequencing a LI-WGS library compared with a SI-WGS library in a single flowcell lane, 93× mapped physical coverage is achieved with the LI-WGS library, whereas only 38× is achieved with a SI library in a single flowcell lane (Figure 4A and B). It was observed that a higher molarity of library is needed for sequencing LI-WGS libraries compared with SI libraries. Based on several tests, we found that 18–19 pM, as quantified by Qubit, is an appropriate amount of LI library to load onto a single lane of a v3 HiSeq flowcell to achieve approximately at least 80% Q30. It was also expected that the size of individual clusters may be larger for LI-WGS libraries. However, comparison of thumbnail images of clusters from the LI- and SI-WGS libraries does not show a visible difference in cluster size (Figure 4A and B). Overall, although we saw minor differences in GC dropout, GC normalized coverage, cluster efficiency and Q30 scores, we did not identify any major changes with respect to cluster sizes.

### Comparison of SI- and LI-WGS using patient samples

To evaluate the utility and feasibility of LI-WGS in actual patient samples, we next performed both SI- and LI-WGS on DNA from fresh frozen tumor and whole blood samples from three separate cancer patients. Patient 1 had metastatic basal cell carcinoma of the skin, patient 2 had metastatic papillary renal cell carcinoma and patient 3 had metastatic bronchial neuroendocrine cancer. For LI-WGS, we generated tumor and normal libraries for each patient with insert sizes ranging from ∼800–900 bp long for final library lengths of ∼1000 bp. We additionally generated SI-WGS libraries with approximate insert sizes ranging from 300–350 bp for final library lengths of ∼400–450 bp. PE sequencing for ∼2 × 100 read lengths was performed for all libraries. LI libraries were each sequenced in single lanes, whereas SI libraries were sequenced across 4–5 lanes (Table 2). Detailed information on the protocol used is described in the Methods section. Sequencing metrics are listed in Table 2.

**Table 2.** Sequencing metrics of SI- and LI-WGS libraries for patients 1, 2 and 3

Metric	Patient 1				Patient 2				Patient 3			
	SI		LI		SI		LI		SI		LI	
Total amount of data generated (GB)	275.6		73.6		285.3		67.4		341.7		74.2	
Q30 data generated (GB)	196.5		60.9		261.8		54.2		307.1		60.9	

	Normal	Tumor	Normal	Tumor	Normal	Tumor	Normal	Tumor	Normal	Tumor	Normal	Tumor

Number of flowcell lanes sequenced	5	5	1	1	4	4	1	1	4	4	1	1
Read length	102	102	101	101	104	104	101	101	104	104	101	101
Average cluster density (K/mm^2^)	958.4		756.0		705.9		712.5		819.3		753.5	
Average PF cluster density (%)	65.0		84.4		88.0		82.5		92.1		85.3	
Total number of reads	1 482 053 066	1 214 032 574	330 467 210	338 562 714	1 512 625 416	1 231 472 418	289 650 634	311 034 636	1 665 626 188	1 617 770 394	402 134 806	270 012 982
Total number of mapped reads	1 387 438 027	1 110 401 730	309 910 146	318 045 021	1 426 314 916	1 158 550 623	270 915 362	289 775 606	1 574 312 575	151 7223 113	376 993 087	253 457 102
% mapped reads	93.62	91.46	93.78	93.94	94.29	94.08	93.53	93.17	94.52	93.78	93.75	93.87
Average mapped sequence coverage^a^	45.11	36.10	9.98	10.24	47.28	38.41	8.72	9.33	52.19	50.30	12.14	8.16
Average mapped physical coverage^a^	144.48	116.03	83.86	86.20	131.40	108.19	73.38	78.33	146.37	140.13	108.28	72.58

^a^Sequence and physical coverages were calculated using all data generated. SI libraries were sequenced across five flowcell lanes, whereas LI libraries were sequenced across one flowcell lane.

Read alignment was performed with Burrows–Wheeler Alignment against the human reference genome (build 37). Using SBS technology (9) and 2 × 83 bp read lengths for LI-WGS libraries and 2 × 100 bp read lengths for SI-WGS libraries, we generated over 10.6 trillion total reads across all three patients and across both WGS types. For the SI whole genomes, we generated average mapped sequence coverages ranging from 36 × to 52× (mean = 45×), and average mapped physical coverages ranging from 108× to 146× (mean = 131×). For the LI genomes, we generated average mapped sequence coverages ranging from 8× to 12× (mean = 10×), and average mapped physical coverages ranging from 72× to 108× (mean = 84×). Coverage differences between the two library types are because of the different number of lanes in which libraries were sequenced and the read lengths used for each library type.

We evaluated several library and sequencing metrics, including the percentage of PCR duplicate reads and GC dropout. No significant differences were observed with respect to percentage of duplicates in the LI and SI libraries. The SI libraries had an average percent duplicate rate of 4.53, whereas the LI libraries had an average of 4.32. No significant differences were also observed when evaluating the extent of GC dropout and median GC normalized coverage in each of the two types of libraries (Student’s *t*-test *P*-values of 0.46 and 0.82, respectively). We did observe a difference between LI and SI libraries with respect to AT dropout (Student’s *t*-test *P* value of 0.02), but the means for the LI and SI groups remain low (LI mean = 2.35, SI mean = 1.40) to indicate an overall low level of dropout in AT rich regions.

To compare copy number and translocation detection analyses, we used SAMtools to randomly select 250 million mapped reads from each data set as 4–5 times more sequencing was performed for SI libraries and because 250 million reads can be generated from a single HiSeq flowcell lane, which represents our design of sequencing an LI library in one lane. This normalization allows us to thus assume that the same amount of sequencing was performed for both SI and LI libraries such that the sequence coverages across each data set are similar. Both copy number and translocation detection analyses were then performed on each normalized data set. Metrics and results from analyses on normalized bam’s are listed in Table 3. Percent tumor cellularity for patient 3’s tumor is not known but the tumor cellularities for patients 1 and 2 were both 50%. Assuming a minimum of 10 anomalous reads required for detection, power calculations were performed for patients 1 and 2 to determine the power for identifying CNVs and translocations. For patients 1 and 2, the power for detecting events in LI data is ∼60–80% greater than the power for detecting events in SI data. If we assume 50% tumor cellularity for patient 3, the power of detecting an event is 0.48 in SI data and 0.87 in LI data.

**Table 3.** Analysis metrics of SI- and LI-WGS libraries for patients 1, 2 and 3

Metric	Patient 1				Patient 2				Patient 3			
	SI		LI		SI		LI		SI		LI	
	Normal	Tumor	Normal	Tumor	Normal	Tumor	Normal	Tumor	Normal	Tumor	Normal	Tumor
Number of tumor cellularity	n/a	50	n/a	50	n/a	50	n/a	50	n/a	n/a	n/a	n/a
Median insert size	328.00	330.00	865.00	861.00	285.00	293.00	852.00	860.00	274.00	275.00	901.00	901.00
Mean insert size	326.70	327.81	848.90	850.22	289.01893	292.970983	849.78	848.03	291.669719	289.751541	901.04	898.37
Insert size standard deviation	29.91	32.80	113.24	100.82	45.67	50.13	110.67	135.52	57.61	56.75	108.42	117.62
Average mapped sequence coverage	8.13	8.13	8.05	8.05	8.29	8.29	8.05	8.05	8.29	8.29	8.05	8.05
Average mapped physical coverage	26.04	13.26.13	67.66	67.76	23.03	23.35	67.72	67.58	23.24	23.09	71.81	71.59
Power to detect event	0.52		0.85		0.48		0.86		n/a		n/a	
GC dropout	5.76	7.46	4.45	5.25	2.74	2.69	3.86	4.40	2.73	2.76	5.32	4.95
AT dropout	2.02	2.78	2.15	2.58	0.98	0.86	2.07	2.17	0.87	0.92	2.71	2.44
Median GC normalized coverage	0.69	0.70	0.81	0.79	0.88	0.90	0.84	0.82	0.86	0.86	0.79	0.79
Total number reads	250 023 370	250 031 044	250 027 715	250 019 339	249 978 232	249 998 124	250 002 762	250 009 726	250 002 357	249 991 693	250 009 553	250 001 078
Number of somatic translocations	4		16		3		5		3		15	
Number of somatic translocations detected that affect a COSMIC gene	0		0		0		0		0		1	
Total number of common translocations	3				0				0			
Number of CNVs identified	48		4		2		0		0		2	
Number of genes affected by CNVs	752		12		0		0		0		12	
Number of COSMIC genes affected by CNVs	16		0		0		0		0		2	
Total number of common genes affected by CNVs	11				0				0			

SI- and LI-WGS bam files were each randomly normalized to ∼250 million mapped reads using SAMtools to allow for a direct comparison across SI and LI data sets.

^a^Power was calculated assuming that a minimum of eight anomalous read pairs are required for detection. Because the tumor cellularity of patient 3 is not known, power calculations were not performed. n/a (not available).

### Copy number analysis

We performed genome-wide CNV detection on each set of patient data. Plots from each analysis are shown in Supplementary Figure S2 and summary results are shown in Table 3. Overall 56 CNVs were identified (Supplementary Table S1). Events that affect COSMIC (Catalogue of Somatic Mutations in Cancer) (10) genes are listed in Table 4. No CNVs were identified for patient 2 in LI data and for patient 3 using SI data. CNVs were defined as having log2 ratios with an absolute tumor/normal ratio of at least 0.75.

**Table 4.** CNVs affecting COSMIC genes identified using SI and LI data

Patient	Library	Chr.	Location	CNV	Length (bp)	Log2 fold	Affected COSMIC genes
1	SI	3	51996100:52507500	Loss	511 400	−0.819	BAP1
1	SI	9	136216500:137456500	Loss	1 240 000	−0.819	BRD3
1	SI	16	88359000:89200400	Loss	841 400	−1.127	CBFA2T3
1	SI	8	38276300:38441900	Loss	165 600	−0.819	FGFR1
1	SI	19	358300:8783100	Loss	8 424 800	−1.234	FSTL3
1	SI	19	358300:8783100	Loss	8 424 800	−1.234	GNA11
1	SI	19	358300:8783100	Loss	8 424 800	−1.234	MLLT1
1	SI	12	56035700:57163000	Loss	1 127 300	−0.789	NACA
1	SI	8	70884500:71036600	Loss	152 100	−0.819	NCOA2
1	SI	22	30055000:30229500	Loss	174 500	−0.819	NF2
1	SI	9	137928600:140766000	Loss	2 837 400	−0.789	NOTCH1
1	SI	9	133827500:133983900	Loss	156 400	−0.819	NUP214
1	SI	19	358300:8783100	Loss	8 424 800	−1.234	SH3GL1
1	SI	19	358300:8783100	Loss	8 424 800	−1.234	STK11
1	SI	19	358300:8783100	Loss	8 424 800	−1.234	TCF3
1	SI	1	798600:3766500	Loss	2 967 900	−0.789	TNFRSF14
3	LI	3	186450400:187448100	Loss	997 700	−0.912	BCL6
3	LI	3	186450400:187448100	Loss	997 700	−0.912	EIF4A2

Chr=chromosome

To evaluate the level of noise and variability in the CNV data, we determined the DLRS for each data set. This measurement is used as a standard in evaluating consistency in log ratio array comparative genomic hybridization data for CNV detection and is thus applied here to evaluate data quality. Higher values are indicative of increased noise and less accuracy in CNV detection. Results are shown in Supplementary Table S2. Overall, the DLRS values are lower for the LI libraries compared with SI libraries for each patient. Additionally, the patient 1’s SI data demonstrated the highest whole genome DLRS of 0.117, which correlates with the higher level of noise that is observed in the CNV plot (Supplementary Figure S2A). This increased noise further correlates with the high number of CNVs identified in the patient 1’s SI data and not in the patient 1’s LI data.

To validate CNVs, we performed CNV detection on whole exome data generated from the same paired tumor and normal samples that were whole genome sequenced for each patient. We used this approach since the 1000 Genomes Project demonstrated the feasibility of performing CNV detection using exome data (11). We generated over 735 million reads with mean target coverages ranging from ∼59×–171×. Metrics and CNV analysis results are listed in Supplementary Table S3. We did not identify any genic CNVs in patients 2 and 3, but identified four genic CNVs in patient 1. The absence of genic CNVs in patient 2 in both SI and LI data correlates with the absence of CNVs in the exome data. For patient 1, of the four exome CNVs, one of these events overlap with a CNV identified in patient 1’s LI data and another event overlaps with a CNV identified in patient 1’s SI data. For patient 3, genic CNVs were only identified in LI data but these events were not identified in exome data. We also evaluated the DLRS on the exome data sets (Supplementary Table S2)—exome data for all three patients had DLRS values >0.1, and with the exception of patient 1’s SI data, the exome DLRS values were all higher than SI and LI data. The high DLRS values for all three patients’ exome data indicate that increased noise may have affected CNV detection and that lower CNV detection accuracy is associated with these data. Patient 1’s exome data also had the highest DLSR across both exome and WG sequencing (0.142), and thus suggests decreased accuracy in CNV detection in this patient’s exome data.

### Translocation detection

We identified inter- and intra-chromosomal translocations in each tumor genome that did not have any supporting germline reads. These events were individually evaluated in Integrated Genomics Viewer, and final results from these analyses are listed in Table 3. For each patient, a larger number of translocations were identified using LI libraries as compared with SI libraries. No overlapping somatic translocations were identified across SI and LI libraries for patients 2 and 3, but three overlapping events were identified in patient 1. Table 5 lists all identified translocations in genic regions. Results were compared against COSMIC. Only one identified translocation affected a COSMIC gene (LPP in patient 3’s LI data). Based on availability of samples, we next performed validation of selected translocations using PCR and Sanger sequencing for patient 1 to compare events identified through SI and LI sequencing. Asterisked genes in Table 5 indicate the translocations that were validated. Overall we confirmed the presence of one event that was identified in both the SI and LI data (affecting ERC2 and LIN7A), and also confirmed the presence of an LI event that was not identified in the SI data (affecting GDA and chrX).

**Table 5.** Genic translocations identified using SI and LI data

Patient	Library	Breakpoint location	Affected genes
1	SI	−:7:133311200|−:6:118209600	EXOC4
1	SI	−:3:55788800|−:12:81208800	ERC2, LIN7A^a^
1	LI	+:18:29128000|+:3:150368000	DSG2
1	LI	+:6:125820000|+:7:121984000	CADPS2
1	LI	+:9:74810000|+:X:11950000	GDA^a^
1	LI	−:3:150370000|−:18:29126000	DSG2
1	LI	+:12:81208000|+:3:55788000	ERC2, LIN7A^a^
1	LI	+:X:11952000|+:9:74808000	GDA^a^
1	LI	−:6:118210000|−:7:133310000	EXOC4
1	LI	+:14:89290000|+:17:78272000	TTC8, RNF213
1	LI	+:8:140172000|+:9:116200000	C9orf43
1	LI	−:4:91966000|−:11:83130000	FAM190A
2	SI	−:7:153790400|−:7:149700000	DPP6
2	LI	+:4:130930800|+:12:65817400	MSRB3
2	LI	−:5:43080400|−:5:43269600	NIM1
2	LI	+:7:34837000|+:11:57763200	NPSR1
3	SI	+:12:9576000|+:12:9460000	DDX12P, LOC642846
3	LI	+:3:11258400|+:3:188188800	HRH1, LPP
3	LI	+:3:173983200|+:3:187771200	NLGN1
3	LI	−:11:60480000|−:7:25058400	MS4A8B

^a^Validated by PCR and Sanger sequencing.

## DISCUSSION

With the rapid development of sequencing technologies, next-generation sequencing has become a valuable approach to characterize cancer genomes. As algorithms and technologies continue to evolve, we are tasked with identifying the most robust strategies to ascertain cancer genomes. Although exome sequencing and RNAseq support the identification of point mutations and expression changes, there remains a need to identify a cost-effective approach to identifying translocations and CNVs and that does not require 30× coverage. In the MI-ONCOSEQ study, Rowchowdhury *et al.* (3) previously demonstrated the use of shallow SI-WGS to 5–15× coverage, along with exome and RNAseq, to evaluate tumor genomes with the goal of identifying actionable events in advanced stage cancer patients. They were able to use shallow SI-WGS to identify copy number alterations and structural rearrangements, exome sequencing to identify point mutations and RNAseq to identify expression changes. Although using shallow SI-WGS to identify larger somatic alterations is feasible, the inherent nature of SI-WGS, particularly with shallow coverage, directly decreases our ability to confidently identify larger somatic events because of the lower level of physical coverage that is achieved. We show in this study that shallow sequencing of longer inserts increases our power for translocation and CNV detection over shallow SI-WGS.

We first performed *in silico* analyses to evaluate the utility of LI-WGS. Results from our analyses demonstrate that sequencing LIs compared with SIs increases physical coverage such that less sequencing is needed for LIs to achieve a target physical coverage. We also show that LI-WGS increases our power to detect a heterozygous event even when using shorter read lengths. These analyses thus illustrate the strength of sequencing LIs over SIs when the goal is to identify larger somatic events that are not captured through exome sequencing. An additional advantage is that the use of LI libraries improves our ability to align sequence data against the human reference genome because we acquire information on a larger genomic region. The protocol we developed also requires 1.1 µg of input DNA, whereas mate pair protocols require microgram amounts of DNA. Furthermore, the protocol we describe here is more user-friendly compared with mate pair protocols because mate pair protocols require all the steps in our approach as well as additional procedures including circularization and linearization of the DNA, multiple enzymatic digestions and purification steps. The protocol we describe here requires ∼1.5 days, whereas standard mate pair protocols require 3 days. Lastly, our protocol uses sonication for fragmentation, and thus does not require trimming of transposase footprints post-sequencing. Overall, we can simultaneously reap the benefits of its application and decrease costs as Illumina’s standard mate pair preparation for a single library is 7× the cost of generating a LI library using Illumina’s TruSeq DNA Sample Prep Kit.

A few caveats of LI-WGS are that it requires the availability of biopsies with sufficient tumor cellularity and also requires that sufficient high quality DNA be isolated from these biopsies. Lower cluster densities are also achieved with LI-WGS but because less sequencing is needed for LI-WGS, this difference does not inhibit its application. Although LI-WGS improves our ability to detect CNVs and translocations, improved algorithms for detection of structural variants are still needed. Numerous bioinformatics tools, including DELLY (12), clipping reveals structure (CREST) (13), BreakDancer (14) and others (15,16), have been developed for structural variant detection. Downstream testing of currently available algorithms on LI-WGS libraries is warranted to further optimize structural variant detection. We also note that the cost of shallow LI-WGS is the same cost as shallow SI-WGS but the increased power in detecting events using LI-WGS is a significant benefit to take advantage of.

We additionally demonstrate the application of LI-WGS, compared with SI-WGS, to three separate cancer patients for identification of somatic CNVs and translocations and performed validation on both types of events. The high DLRS of the exome validation data across all three patients indicates a high level of noise in the exome data, and thus reflects the differences in identified CNVs that were seen across the exome and SI and LI data sets. Although this finding emphasizes the need for improved algorithms for identifying CNVs in non-WGS assays, two events were validated in patient 1, and the absence of events in patient 2 WGS data correlates with the absence of events in patient 2’s exome validation data. Overall, the LI data also had a lower DLRS compared with the SI data for each patient, and thus emphasizes the decrease in noise and increase in CNV detection accuracy in LI data. Power calculations for patients 1 and 2, for whom we know the tumor cellularities, also show that our power for detecting events is 60–80% greater when using LI data over SI data. While knowledge of tumor cellularity improves interpretation of LI-WGS results, we show the feasibility and utility of LI-WGS. In conclusion, LI-WGS represents a single assay that can be used to simultaneously identify CNVs and translocations, results in less noise for CNV detection, increases our power to detect changes due to the higher physical coverage that is achieved and is more cost-effective and user-friendly as modifications need only be made to an established library generation protocol.

As the research community continues to enable current technologies to understand cancers and other diseases, we are tasked with the challenges of fine tuning both wet lab and bioinformatics analyses to improve genomic analyses and characterizations. As such, identifying and applying the most cost-effective and robust approaches to evaluating cancer genomes are needed. In this study, we illustrate the feasibility of LI-WGS as well as its utility in detection of somatic copy number changes and translocations. This approach is also not limited to cancer and may be applied to other diseases. By optimizing an established WGS library preparation protocol, we show that we can improve our ability to detect structural variants without performing an overhaul of current approaches. Continued improvements in genomic analyses will strengthen the foundation for personalized medicine and set the stage for developing and pinpointing efficacious treatments for patients.

## ACCESSION NUMBERS

The NCBI (National Center for Biotechnology Information) dbGaP study accession number for patient data from this study is phs000646.v1.p1 (http://www.ncbi.nlm.nih.gov/projects/gap/cgi-bin/study.cgi?study_id=phs000646.v1.p1).

## SUPPLEMENTARY DATA

Supplementary Data are available at NAR Online.

## FUNDING

Funding for open access: Institutional funds.

*Conflict of interest statement*. None declared.

## Supplementary Material

Supplementary Data
