# Supplementary material for: Long insert whole genome sequencing for copy number variant and translocation detection
Source: Nucleic Acids Res. 2013 Sep 25;42(2):e8. doi: 10.1093/nar/gkt865 (PMC3902897; doi:10.1093/nar/gkt865)
Supplement: Supplementary Data [file supp_42_2_e8__index.html]

Long insert whole genome sequencing for copy number variant and translocation detection — Long insert whole genome sequencing for copy number variant and translocation detection — Supplementary Data 

# Long insert whole genome sequencing for copy number variant and translocation detection

## Supplementary Data

files

**Files in this Data Supplement:**

- Supplementary Data - pdf file
