# Supplementary material for: Long insert whole genome sequencing for copy number variant and translocation detection
Source: Nucleic Acids Res. 2013 Sep 25;42(2):e8. doi: 10.1093/nar/gkt865 (PMC3902897; doi:10.1093/nar/gkt865)

Supplementary Table 1.

All CNVs (|log2 ratio| > 0.75) identified across all patients and library types are listed.

Patient	Library	Location	Log2 ratio
1	SI	chr1: 148638400-149778300	-1.275
1	SI	chr7: 150580200-151116800	-1.234
1	SI	chr19: 358300-8783100	-1.234
1	SI	chr2: 241558600-242208800	-1.127
1	SI	chr16: 88359000-89200400	-1.127
1	SI	chr11: 2745200-2990200	-1.012
1	SI	chr12: 34406300-34563100	-1.012
1	SI	chr6: 84592400-84814000	-0.918
1	SI	chr8: 141483200-141684000	-0.918
1	SI	chr3: 48536000-48729700	-0.905
1	SI	chr3: 50305600-50621800	-0.905
1	SI	chr3: 52785200-52932200	-0.905
1	SI	chr8: 21559500-21749000	-0.905
1	SI	chr8: 22507800-22612300	-0.905
1	SI	chr8: 46951300-47281400	-0.905
1	SI	chr8: 126349400-126794700	-0.905
1	SI	chr8: 144194000-145920600	-0.905
1	SI	chr12: 132864800-133397100	-0.905
1	SI	chr2: 218784800-218832300	-0.819
1	SI	chr3: 51996100-52507500	-0.819
1	SI	chr8: 1415600-2095400	-0.819
1	SI	chr8: 21933200-22201500	-0.819
1	SI	chr8: 23060000-23692200	-0.819
1	SI	chr8: 38276300-38441900	-0.819
1	SI	chr8: 70884500-71036600	-0.819
1	SI	chr8: 140574400-141060100	-0.819
1	SI	chr9: 127157700-127200500	-0.819
1	SI	chr9: 129332200-129544300	-0.819
1	SI	chr9: 133827500-133983900	-0.819
1	SI	chr9: 136216500-137456500	-0.819
1	SI	chr10: 74007900-74184600	-0.819
1	SI	chr10: 80812700-80997300	-0.819

1	SI	chr10: 103787000-104009400	-0.819
1	SI	chr12: 124722800-126026200	-0.819
1	SI	chr16: 87602700-87918900	-0.819
1	SI	chr18: 11700-153900	-0.819
1	SI	chr22: 30055000-30229500	-0.819
1	SI	chr1: 798600-3766500	-0.789
1	SI	chr3: 46941200-47020000	-0.789
1	SI	chr3: 126649200-126839900	-0.789
1	SI	chr9: 130528800-131209000	-0.789
1	SI	chr9: 137928600-140766000	-0.789
1	SI	chr10: 88352200-88550400	-0.789
1	SI	chr12: 56035700-57163000	-0.789
1	SI	chr12: 56035700-57163000	-0.789
1	SI	chr12: 130879100-131211800	-0.789
1	SI	chr15: 25381700-25399200	-0.789
1	SI	chr17: 4327500-4985600	-0.789
1	LI	chr6: 84606600-84806100	-0.98
1	LI	chr8: 143513900-143712200	-0.803
1	LI	chr17: 80832100-80084000	-0.803
1	LI	chr8: 145613600-145709200	-0.791
2	SI	chr2: 89563000-91640400	-0.84
2	SI	chr8: 39175800-39408300	-0.755
3	LI	chr3: 186450400-187448100	-0.912
3	LI	chr16: 33842100-34871800	-0.764

Supplementary Table 2. Derivative log ratio spread (DLRS) analysis

DLRS was calculated for SI and LI data sets as well as for exome validation data for each patient. SI and LI analyses were performed on normalized bam's.

	**Patient 1**			**Patient 2**			**Patient 3**		
	SI	LI	Exome	SI	LI	Exome	SI	LI	Exome
DLRS	0.117	0.096	0.142	0.084	0.082	0.1226	0.083	0.078	0.108

Supplementary Table 3. Exome sequencing metrics & CNV detection

Exome sequencing was performed on the same tumor/normal pairs that were whole genome sequenced for each patient to validate CNVs.

	Patient 1		Patient 2		Patient 3	
Total amount of data generated (GB)	13.3		27.6		29.2	
Q30 data generated (GB)	11.1		24.8		23.1	
Read length	2x101		2x101		2x101	
Total #reads	125473318		224890888		301471184	
	**Normal**	**Tumor**	**Normal**	**Tumor**	**Normal**	**Tumor**
Total #mapped reads	53899773	67064679	168269051	152581663	149798936	143945852
%mapped reads	96.28	96.51	98.8	98.7	97.18	97.7
Average target coverage	59.57	75.04	143.20	113.96	152.30	171.20
# CNVs identified	4		0		0	

Supplementary Table 4. CNVs identified in exome sequencing data

2 of 4 genic CNVs identified in exome data overlap with CNVs identified in patient 1 LI data. CNV locations for LI and SI data are shown. Y=yes, N=no

Patient	Log2 ratio	Location	In whole genome data?
1	0.792	chr9:15433600-17332500	N
1	-0.850	chr17:79517200-80048500	Y (LI; chr17:80832100-80084000)
1	-1.286	chr19:7869-3633300	Y (SI; chr19:358300-8783100)
1	-0.816	chr19:4268500-4860000	N

Supplementary Figure 1. Comparison of sequencing required to achieve a target physical coverage

A priori analyses were performed to compare the number of reads required using SI (300bp) or LI (900bp) libraries to achieve a target physical coverage.

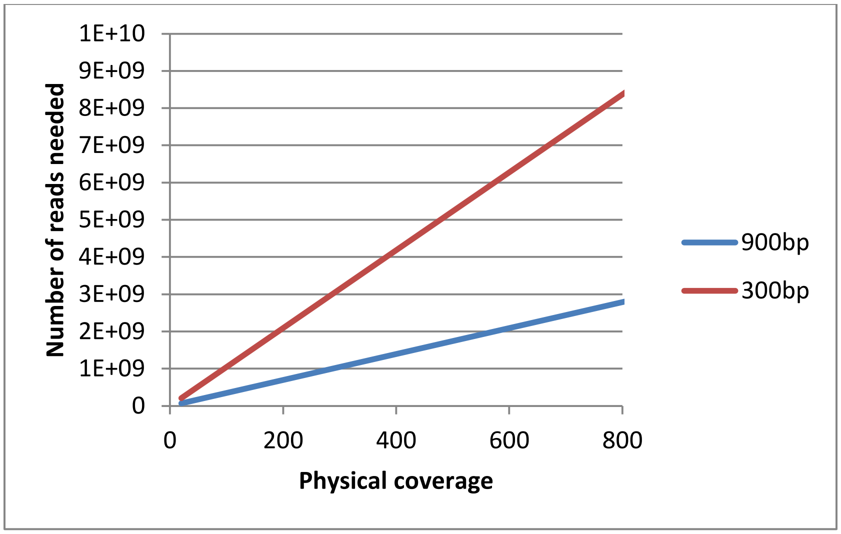

Supplementary Figure 2. Copy number analysis

Copy number change plots are shown for both SI-WGS and LI-WGS normalized data for each of 3 patients: (A) patient 1 SI-WGS, (B) patient 1 LI-WGS, (C) patient 2 SI-WGS, (D) patient 2 LI-WGS, (E) patient 3 SI-WGS, (F) patient 3 LI-WGS. Results are organized by chromosome. Red demarcates indicate copy number gains and green demarcates copy number losses (|log2 ratio| > 0.75).

A) Patient 1 SI-WGS

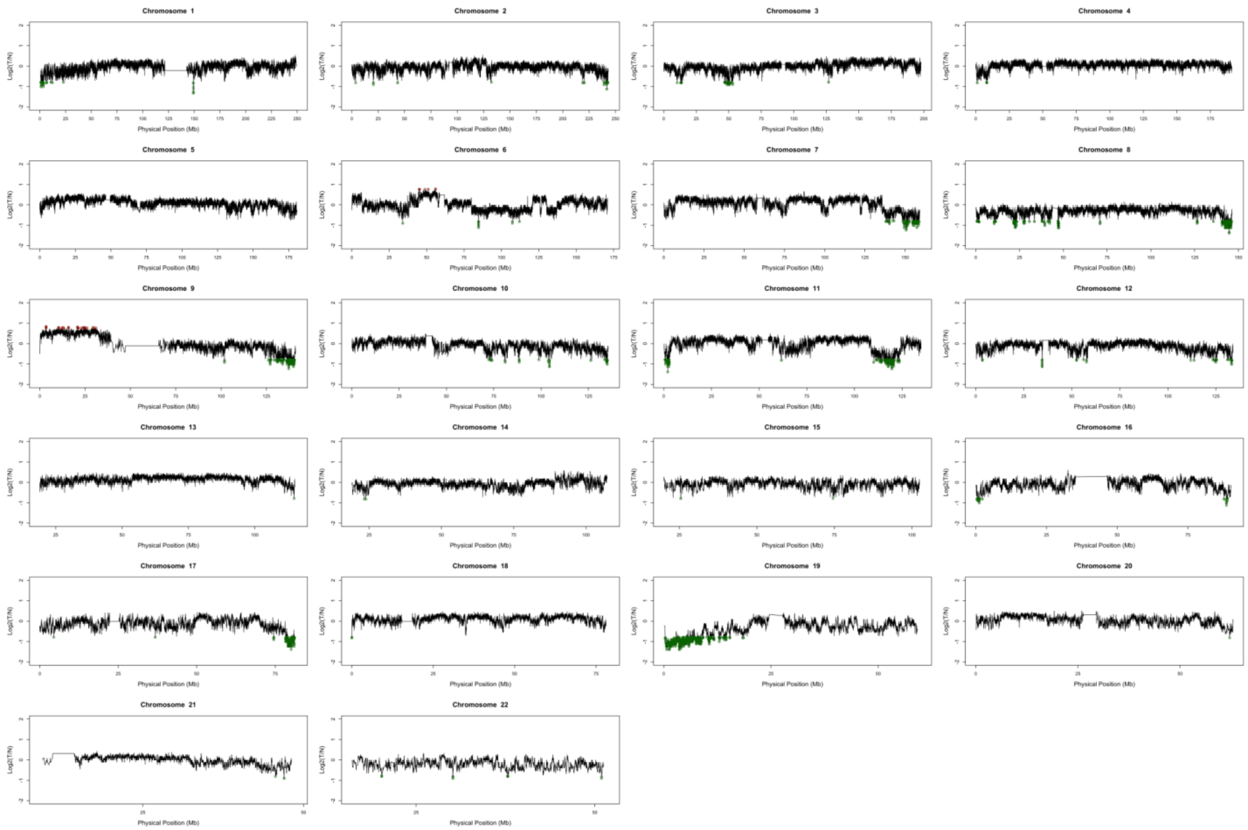

B) Patient 1 LI-WGS

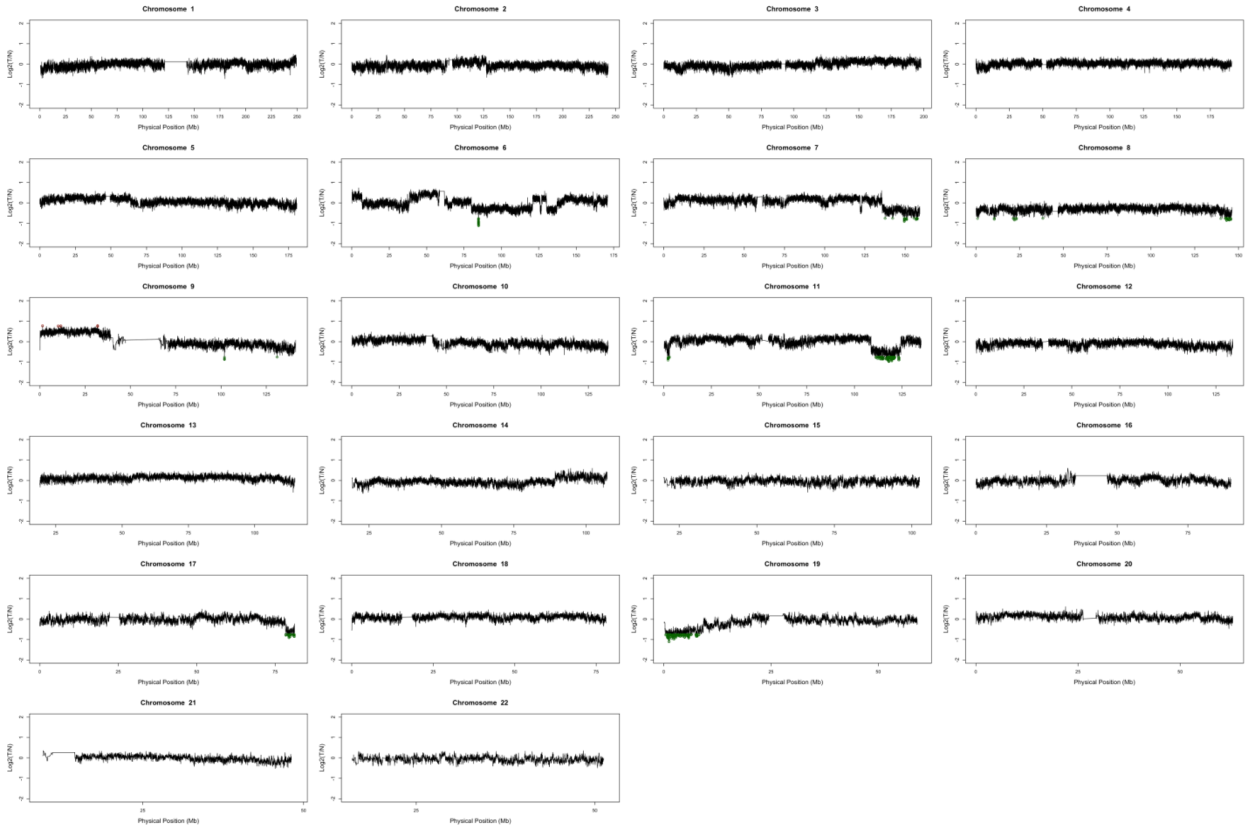

C) Patient 2 SI-WGS

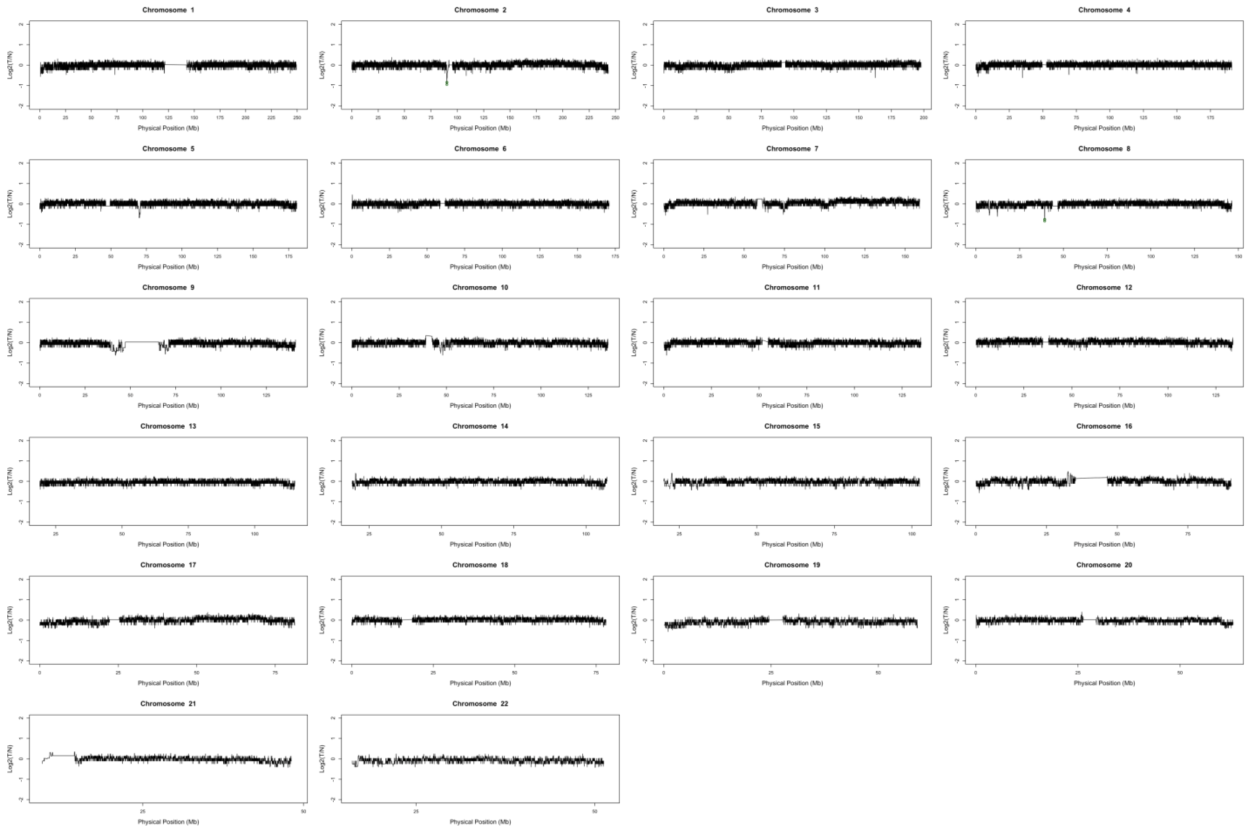

D) Patient 2 LI-WGS

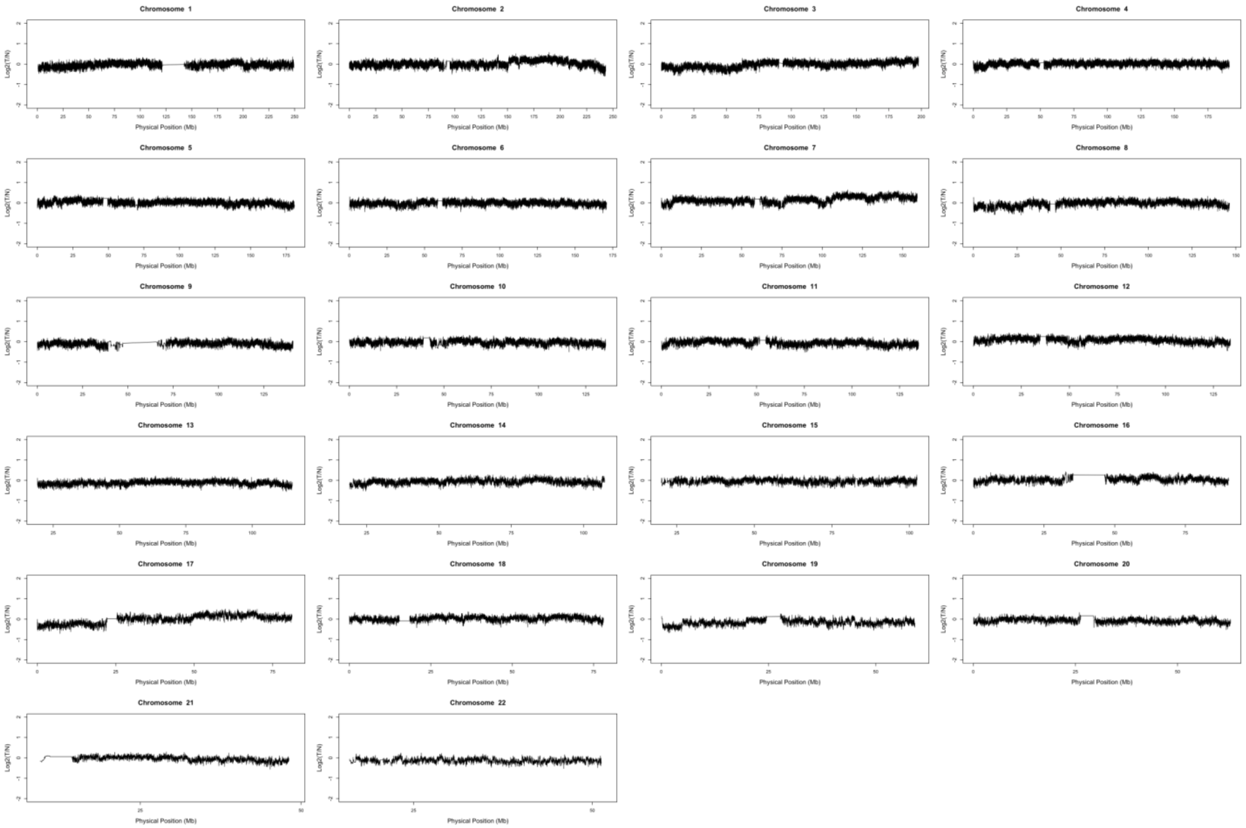

E) Patient 3 SI-WGS

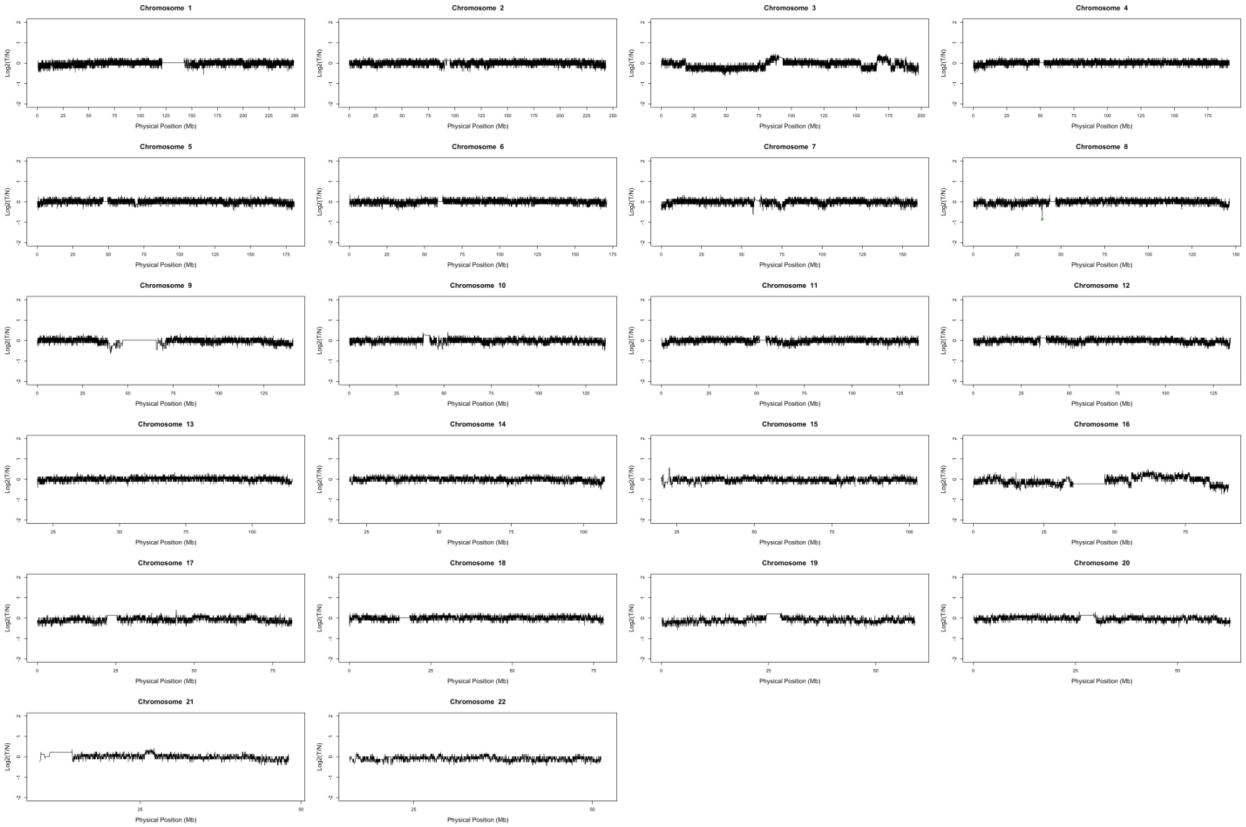

F) Patient 3 LI-WGS

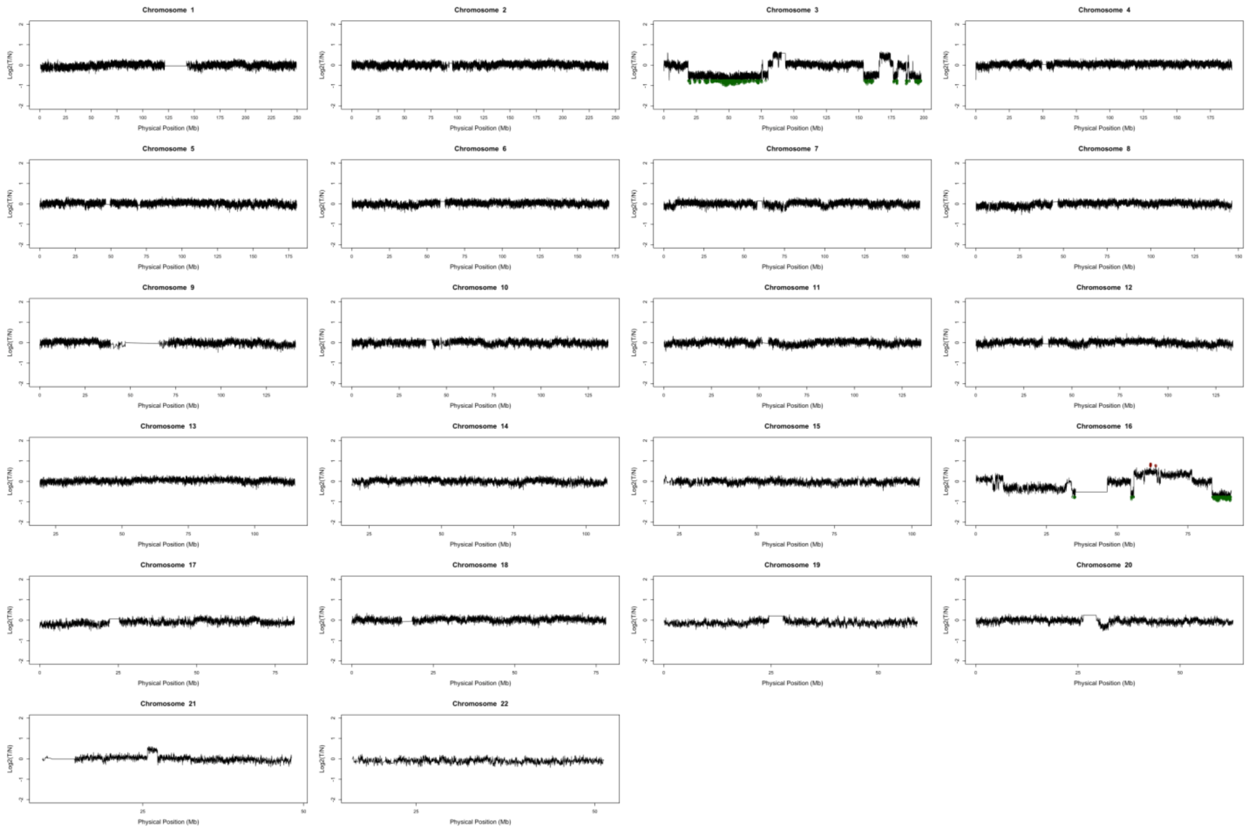

Supplement: Supplementary Data [file supp_gkt865_nar-01794-met-g-2013-File006.pdf]
